# Glycoprotein Hormone Receptor Knockdown Leads to Reduced Reproductive Success in Male *Aedes aegypti*

**DOI:** 10.3389/fphys.2019.00266

**Published:** 2019-03-19

**Authors:** David A. Rocco, Ana S. G. Garcia, Elton L. Scudeler, Daniela C. dos Santos, Rafael H. Nóbrega, Jean-Paul V. Paluzzi

**Affiliations:** ^1^Department of Biology, York University, Toronto, ON, Canada; ^2^Department of Morphology, Institute of Biosciences of Botucatu, São Paulo State University (UNESP), Botucatu, Brazil; ^3^Electron Microscopy Center, Institute of Biosciences of Botucatu, São Paulo State University (UNESP), Botucatu, Brazil

**Keywords:** leucine-rich repeat-containing G protein-coupled receptor 1 (LGR1), glycoprotein hormone receptor, GPA2/GPB5, mosquito, spermatogenesis, centriole adjunct

## Abstract

Glycoprotein hormone receptors mediate a diverse range of physiological functions in vertebrate and invertebrate organisms. The heterodimeric glycoprotein hormone GPA2/GPB5 and its receptor LGR1, constitute a recently discovered invertebrate neuroendocrine signaling system that remains to be functionally characterized. We previously reported that LGR1 is expressed in the testes of adult *Aedes aegypti* mosquitoes, where its immunoreactivity is particularly regionalized. Here, we show that LGR1 immunoreactivity is associated with the centriole adjunct of spermatids and is observed transiently during spermatogenesis in mosquitoes, where it may act to mediate the regulation of flagellar development. RNA interference to downregulate LGR1 expression was accomplished by feeding mosquito larvae with bacteria that produced LGR1-specific dsRNA, which led to defects in spermatozoa, characterized with shortened flagella. LGR1 knockdown mosquitoes also retained ∼60% less spermatozoa in reproductive organs and demonstrated reduced fertility compared to controls. To date, the endocrine regulation of spermatogenesis in mosquitoes remains an understudied research area. The distribution of LGR1 and detrimental effects of its knockdown on spermatogenesis in *A. aegypti* indicates that this heterodimeric glycoprotein hormone signaling system contributes significantly to the regulation of male reproductive biology in this important disease-vector.

## Introduction

The mosquito *Aedes aegypti* serves as a vector for a variety of pathogens causing diseases including Zika, yellow fever, chikungunya and dengue virus, the latter of which is the most widespread arbovirus disease in humans infecting nearly 400 million people annually (Bhatt et al., 2013). Currently, promising control strategies that aim to curb viral transmission by mosquitoes target their reproductive physiology. For example, the classical sterile insect technique (SIT), which involves the mass production and release of sterilized lab-reared male insects into field populations, has proven effective in the elimination of the New World screwworm *Cochliomyia hominivorax* from the southern United States, Central America, Mexico and Panama, and has had success for a number of mosquito species (Benedict and Robinson, 2003; Wyss, 2006; Robinson et al., 2009; Oliva et al., 2014). A second promising control strategy to reduce disease transmission by mosquitoes involves introducing the bacterium *Wolbachia* to mosquito populations, which renders the sperm of infected males incapable of fertilization (LePage and Bordenstein, 2013).

Control methods that target reproduction in disease vectors can be improved with a comprehensive knowledge of their reproductive biology, including its regulation. In *A. aegypti*, spermatogenesis is asynchronous and occurs in a pair of testes that are not always the same size nor in the same state of development (Hutcheson, 1972). Each testis resembles a follicle organized into different compartments called cysts, characterized with an external wall that envelopes numerous germ cells developing in a synchronous fashion (Wandall, 1986; Clements, 2000). From the apex to the base of the testis, cysts progressively increase in size and maturity so that smaller compartments with undifferentiated spermatogonia are found on distal ends, whereas larger cysts containing mature spermatozoa are located at the base (Wandall, 1986; Clements, 2000). As maturation proceeds in the testis, spermatogonia proliferate and differentiate into spermatocytes, which undergo two meiotic divisions to produce spermatids and finally spermatozoa; which are then transported from each testis, via the vas deferens, to a common storage unit called the seminal vesicle. Upon copulation with a female, the seminal vesicle is emptied, and a new wave of spermatogenesis occurs to ensure that a continuous supply of sperm is made available for a subsequent mating (Clements, 2000). While much is known regarding the regulation and control of reproductive processes in female insects including oogenesis, research dedicated to understanding the regulation and control of spermatogenesis by factors including neuropeptides and/or neurohormones remains largely unexplored (Raikhel et al., 2005).

In vertebrates, hormonal regulation of spermatogenesis is governed by the gonadotropins follicle-stimulating hormone (FSH) and luteinizing hormone (LH) (Pierce and Parsons, 1981; Kerr and Sharpe, 1985; Stockell Hartree and Renwick, 1992; Dierich et al., 1998). FSH, LH, thyroid-stimulating hormone (TSH) and chorionic gonadotropin (CG) comprise the classic glycoprotein hormone family, each formed by the heterodimerization of a common alpha subunit and a hormone-specific beta subunit (Pierce and Parsons, 1981). A fifth glycoprotein hormone comprised of two distinct subunits (GPA2/GPB5) called thyrostimulin was discovered and, unlike the classic heterodimeric glycoprotein hormones that are restricted to vertebrate species, homologous GPA2/GPB5 subunit genes have been identified in most bilaterians (Hsu et al., 2002; Nakabayashi et al., 2002; Santos et al., 2011; Rocco and Paluzzi, 2016). Though the exact physiological role of GPA2/GPB5 remains unclear in any given organism, expression studies of the GPA2/GPB5 receptor indicate it may possess a reproductive function in the rat (Sun et al., 2010), mosquito (Rocco et al., 2017), amphioxus (Tando and Kubokawa, 2009) and lamprey (Hausken et al., 2018). In invertebrates, GPA2/GPB5 binds to a receptor called the leucine-rich repeat-containing G protein-coupled receptor 1 (LGR1), which shares ∼50% amino acid sequence homology to the membrane-spanning regions of the classic glycoprotein hormone receptors (Hauser et al., 1998; Nishi et al., 2000; Sudo et al., 2005). In the yellow fever mosquito *A. aegypti*, an investigation on the expression profile of LGR1 revealed transcript expression along with strongly regionalized immunoreactivity in the mosquito testes, suggesting GPA2/GPB5 may be linked to regulating mosquito spermatogenesis (Rocco et al., 2017). In the present study we sought to further delineate the role of LGR1 in *A. aegypti* spermatogenesis by (i) characterizing the distribution of LGR1 expression over the course of spermatogenesis and (ii) using LGR1-targeted RNA interference to examine effects on net spermatozoa output, flagellar length and male fertility.

## Materials and Methods

### *A. aegypti* Colony Rearing

Experimental animals were acquired from colonies raised under conditions described previously (Rocco et al., 2017). To ensure virgin adults, pupae were isolated individually in 25 cm^2^ cell culture flasks (Corning, NY, United States) containing 3 mL of de-ionized water (dH_2_O) and covered with a cotton ball wick soaked in a 10% sucrose solution. Upon emergence, adults were sexed and processed for immunohistochemical and fluorescence *in situ* hybridization experiments.

### Whole Mount Immunohistochemistry

Male reproductive organs (testes, vas deferens and seminal vesicle) and female spermathecae of one-day old virgin adult *A. aegypti* were collected and analyzed for LGR1 expression using immunohistochemical techniques described earlier (Rocco et al., 2017). Primary antibodies included a custom affinity-purified rabbit polyclonal antibody raised against the *A. aegypti* LGR1 protein (0.5 μg ml^-1^, Genscript Inc., Piscataway, NJ, United States) with specificity validated previously (Rocco et al., 2017), and a mouse monoclonal anti-γ-tubulin antibody (1:10, Sigma-Aldrich, Oakville, ON, Canada). Secondary antibodies used included Alexa Fluor 594- or Alexa Fluor 488-coupled goat anti-rabbit Ab (1:200, Invitrogen, Carlsbad, CA, United States), and Alexa Fluor 647-coupled goat anti-mouse Ab (1:200, Invitrogen, Carlsbad, CA, United States). Filamentous actin (F-actin) was visualized using Alexa Fluor 488-conjugated phalloidin (0.165 M, Invitrogen, Carlsbad, CA, United States). For visualization of the mitochondrial derivatives associated with spermatozoa, samples were incubated in MitoTracker Red CMXRos (100 nM, Invitrogen, Carlsbad, CA, United States) in nuclease-free phosphate-buffered saline (PBS) at room temperature (RT) for 2 h prior to fixation. DNA was stained with 4′,6-Diamidino-2-phenylindole dihydrochloride (DAPI) (4 μg mL^-1^, Sigma-Aldrich, Oakville, ON, Canada), and samples were optically sectioned using a Yokogowa CSU-X1 Zeiss Cell Observer Spinning Disk confocal microscope. Images were processed using Zeiss Zen and ImageJ software.

### Fluorescence *in situ* Hybridization

Target cDNA fragments were amplified from previously prepared constructs that contained the *A. aegypti* LGR1 open reading frame (Paluzzi et al., 2014) using gene-specific primers (Supplementary Table S1). PCR amplicons were ligated to pGEM-T-Easy vector (Promega, Madison, WI, United States) and transformed into NEB 5-alpha high efficiency competent *Escherichia coli* (New England Biolabs, Whitby, ON, Canada). The T7 promoter sequence was incorporated onto the 5′ end of the sense and anti-sense strands using modified primers (see Supplementary Table S1), and amplification was carried out using the T7 promoter oligonucleotide and either anti-sense or sense gene-specific primers to create anti-sense or sense strand template cDNA. Base accuracy was verified by Sanger sequencing (The Centre for Applied Genomics, Sick Kids Hospital, Toronto, ON, Canada), and cDNA templates were used to generate *in vitro* transcribed digoxigenin (DIG)-labeled sense and anti-sense RNA probes using the HiScribe T7 High Yield RNA Synthesis Kit (New England Biolabs, Whitby, ON, Canada) and replacing the provided nucleotides with DIG RNA Labeling Mix (Sigma-Aldrich, Oakville, ON, Canada). After overnight synthesis at 37°C, RNA probes were treated with DNAse I (New England Biolabs, Whitby, ON, Canada), electrophoresed on a 1% non-denaturing agarose gel to confirm probe integrity and DNA template removal, and subsequently quantified. The localization of LGR1 transcript in the testes of *A. aegypti* was determined with fluorescence *in situ* hybridization (FISH) following a previously reported protocol (Paluzzi et al., 2008), using 10 ng μl^-1^ sense and anti-sense DIG-labeled RNA probes. Modifications to the protocol reported previously included fluorescent detection with Alexa Fluor 488 tyramide dye diluted (1:100) in amplification buffer (Life Technologies, Eugene, OR, United States) containing 0.0015% H_2_O_2_ and optical sections were performed using a Yokogowa CSU-X1 Zeiss Cell Observer Spinning Disk confocal microscope. Images were processed using Zeiss Zen and ImageJ software.

### Transmission Electron Microscopy

Upon dissecting *A. aegypti* testes in PBS, tissue samples were processed for conventional methods of transmission electron microscopy as previously described (Scudeler et al., 2017). Briefly, tissues were fixed overnight in a 2.5% glutaraldehyde and 4% paraformaldehyde solution in 0.1 M phosphate buffer (pH 7.3) and post-fixed for 2 h in 1% osmium tetroxide. Tissue samples were contrasted with 0.5% uranyl acetate for 2 h, dehydrated in a graded acetone series (50, 70, 90, and 100%), and embedded in Araldite resin. Ultrathin sections were contrasted with uranyl acetate and lead citrate, and analyzed using a Tecnai Spirit transmission electron microscope (FEI Company, Eindhoven, Netherlands).

### Bacteria-Mediated RNA Interference

Double-stranded RNA (dsRNA) target cDNA sequences for *A. aegypti* LGR1 were amplified using primers listed (Supplementary Table S1) using a previous plasmid construct containing the LGR1 open reading frame (Rocco et al., 2017). Primers amplified a select region in the N-terminal ectodomain region (Paluzzi et al., 2014), which did not span the region containing the antigen site for the custom LGR1 antibody used in immunohistochemical experiments nor did it overlap with the primer-binding sites targeted in quantitative RT-PCR (Rocco et al., 2017). The amplified dsRNA template sequence was then cloned into the pGEM-T-Easy sequencing vector (Promega, Madison, WI, United States) and subcloned into the dsRNA-producing plasmid L4440 (Fire et al., 1998; Montgomery et al., 1998), which was a gift from Andrew Fire (Addgene plasmid # 1654). L4440 plasmids containing the LGR1 target sequence or an empty vector (used as a control) was then transformed into RNAse III-deficient HT115 (DE3) *E. coli* bacterial strain provided by the *Caenorhabditis* Genetics Center, which is funded by NIH Office of Research Infrastructure Programs (P40 OD010440). HT115 (DE3) *E. coli* containing the L4440 plasmid with or without the LGR1 target sequence were grown in 5 mL LB media supplemented with ampicillin (100 μg ml^-1^) and tetracycline (10 μg ml^-1^) (LB-Amp-Tet). Overnight cultures were then used to inoculate 100 mL of pre-warmed LB-Amp-Tet with shaking at 37°C and bacterial growth was measured hourly using a Synergy 2 Multi-Mode Microplate Reader (BioTek) until an OD_595_ of 0.7–0.8 was reached. Bacterial cultures were then induced with 0.4 mM ispropyl β-D-1-thiogalactopyranoside (IPTG) to produce dsRNA for 4 h, shaking at 37°C. After IPTG induction, cultures were centrifuged at 5000 rpm for 10 min, and bacterial pellets were resuspended in 1 mL LB media (without antibiotics) and stored overnight at –20°C. The next day, bacteria were thawed, mixed with an equal volume of larval feed (2% liver powder: brewers yeast mixture, 1:1) and used to feed 2nd instar *A. aegypti* larvae which had been starved overnight. Following 48 h of exposure to control bacteria or LGR1 dsRNA-expressing bacteria, larval baths were changed with clean dH_2_O and larvae were fed normal larval feed until pupation. Pupae were kept isolated to ensure emerging adults were virgin for future experiments.

### Evaluation of LGR1 Knockdown Efficiency

#### RT-qPCR

Total RNA was isolated from 4th instar larvae and one-day old virgin adult *A. aegypti* that were fed on *E. coli* expressing control dsRNA (empty L4440) or LGR1 dsRNA using the Monarch Total RNA Miniprep Kit (New England Biolabs, Whitby, ON, Canada) following manufacturer’s instructions that included an on-column DNAse I treatment to remove residual genomic DNA. Total RNA (50 ng) was then used as template for cDNA synthesis using the iScript^TM^ Reverse Transcription Supermix (Bio-Rad, Mississauga, ON, Canada) following recommended guidelines and diluted ten-fold prior to quantitative RT-PCR. LGR1 transcript levels were then quantified using PowerUP^TM^ SYBR^®^ Green Master Mix (Applied Biosystems, Carlsbad, CA, United States) and measured on a StepOnePlus Real-Time PCR System (Applied Biosystems, Carlsbad, CA, United States) following conditions described previously (Gondalia et al., 2016). Primers used to quantify LGR1 transcript abundance overlap exon-exon boundaries (Supplementary Table S1), and data was normalized to the stably expressed reference gene ribosomal protein 49 (GenBank accession: AY539746) according to the ΔΔCt method (Paluzzi et al., 2014). Measurements were taken from a minimum of three independent rounds of bacterial feedings, each consisting of 50 control or dsLGR1-treated larvae, to serve as biological replicates for each developmental stage.

#### Immunofluorescence

LGR1 immunoreactive staining intensity was compared between testes, particularly in cysts containing late-staged spermatids, of one-day old virgin *A. aegypti* that were either fed on *E. coli* expressing control dsRNA (empty L4440) or LGR1 dsRNA. All microscope settings were identical when acquiring images of control and dsLGR1-treated testes and saturation was avoided by setting appropriate acquisition parameters as recommended to improve the accuracy and precision of fluorescence microscopy-based quantitative measurements (Waters, 2009). Using ImageJ software, immunofluorescent images were converted to 8-bit grayscale images, over an intensity scale of 0–255, and the mean gray value from cysts within the testes containing LGR1 immunoreactivity was recorded and compared between treatments. Measurements of LGR1 staining intensity compared isolated control or dsLGR1-treated testes (*n* = 11–13) that served as independent biological replicates. Testes of adult mosquitoes collected for analysis were gathered from multiple batches of bacterially fed larvae.

### Quantitation of Sperm and Flagella Length

Sperm quantitation was performed following a protocol that was previously shown to obtain accurate sperm counts (Ponlawat and Harrington, 2007). Male reproductive organs (testes and seminal vesicle) were isolated in PBS from adult virgin *A. aegypti* that were fed as larvae with either *E. coli* expressing control dsRNA (empty L4440) or LGR1 dsRNA. For each specimen, testes were separated from the seminal vesicle and placed into designated wells of a 24-well tissue culture plate containing 100 μl PBS. Organs were then carefully torn using 0.1 mm insect pins to release sperm. Pins were washed with an excess of 100 μl PBS for a final volume of 200 μl/well/tissue. Upon aspirating each well with a P10 pipette to obtain a homogenous distribution of sperm, 5 μl of PBS from each well was spotted 10 times onto microscope slides, air-dried and fixed with 70% ethanol. After fixation, slides were stained with NucBlue^®^ Cell Stain Ready Probes^TM^ (Life Technologies, Eugene, OR, United States) and elongated nuclei, characteristic of mature spermatozoa, were counted in each 5 μl aliquot. The average number of spermatozoa in a 5 μl droplet was calculated and multiplied by the dilution factor to obtain a measure of the total number of spermatozoa per tissue sample. Total number of spermatozoa from the testes or seminal vesicle were compared between control and LGR1 knockdown treatments (*n* = 17 mosquitoes, collected from multiple batches of bacterially fed larvae). Counts were performed blindly two times to maintain accuracy in sperm quantitation. Flagella associated with mature spermatozoa were visualized by phase-contrast microscopy and lengths were measured using an EVOS FL Auto Imaging System (Life Technologies, Burlington, ON, Canada). The average flagellar length of 60 testis-derived spermatozoa was compared between mosquitoes treated with control or dsLGR1 bacteria (*n* = 14–15 mosquitoes, collected from multiple batches of bacterially fed larvae).

### Mating Assays

One-day-old virgin male *A. aegypti*, fed as larvae on either control or LGR1-dsRNA producing bacteria, were individually transferred into flasks containing a six-day-old untreated virgin female (each flask contained one male per female), and provided a cotton ball wet with dH_2_O to entice a bloodmeal the following day. Individual females were blood fed on the arm of a consenting adult over the mating flask that contained a small opening lined with black aluminum screening. Three days post-bloodmeal, filter paper was provided for eggs to be oviposited, and the eggs from each female were collected and counted 72 h later. Upon semi-desiccating eggs for 48 h, eggs were placed into 100 mL dH_2_O containing 500 μl of larval feed for 3 days, and percent larval hatching was calculated. Each egg batch laid by a given female mosquito served as an independent biological replicate (*n* = 13).

## Results

### *A. aegypti* LGR1 Expression Localizes to the Centriole Adjunct of Spermatids

**FIGURE 1:**
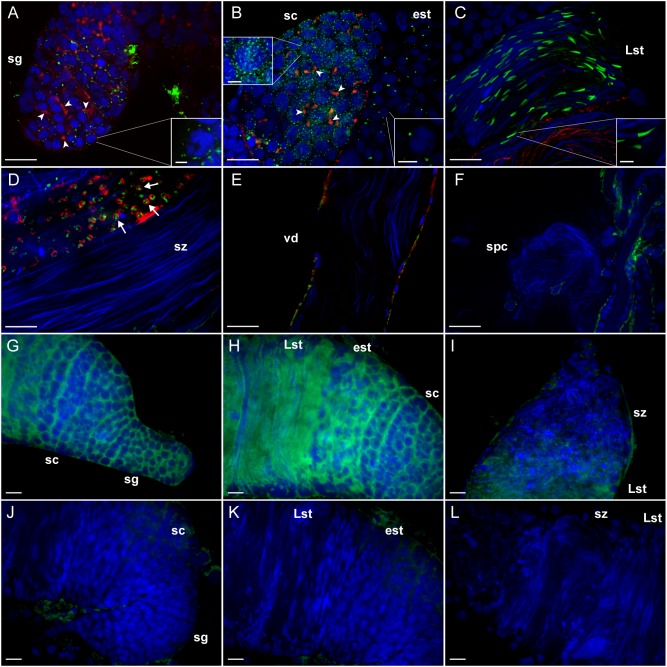
Immunohistochemical localization and transcript detection by fluorescent *in situ* hybridization of LGR1 during spermatogenesis in adult *Aedes aegypti* testes. **(A,B)** LGR1-immunoreactivity (green), which is distinct from phalloidin-labeled filamentous-actin-rich fusomes (red) (arrowheads) and nuclei (blue), is present as dispersed foci within the cytoplasm of cysts containing spermatogonia (sg) that increase in quantity in cysts containing spermatocytes (sc). **(B)** In early staged spermatids (est), LGR1 immunoreactivity is localized to a single spot that is found on one end of each nucleus and **(C)** elongates as nuclear elongation occurs in late-staged spermatids (Lst). **(D)** LGR1 immunoreactivity is encapsulated by phalloidin-labeled filamentous-actin rings (arrows) known as waste bags (Texada et al., 2008) and **(D–F)** is not associated with the nuclei of mature spermatozoa (sz) within the testis base, vas deferens (vd) or female spermatheca (spc). **(G–I)** Digoxigenin-labeled anti-sense probes used to localize LGR1 mRNA-expressing (green) cells detected receptor transcript in all developmental stages except spermatozoa (nuclei, blue). **(J–L)** Though non-specific staining was associated with fat body surrounding testes using sense probes, LGR1 transcript staining was absent in all spermatogenic cells. Scale bars: 20 μm in panels **A–C, E–L**, 10 μm in panel **D** and 5 μm in panels **A–C** insets.

To identify the role of LGR1 in *A. aegypti* spermatogenesis, we first analyzed the subcellular distribution of LGR1 in each developmental stage of spermatogenesis using immunofluorescence microscopy and *in situ* hybridization. Receptor immunoreactivity and transcript were detected in early spermatogenic cells including spermatogonia, spermatocytes and spermatids, but not in mature spermatozoa (Figure 1). In spermatogonia, LGR1 immunoreactivity was detected as dispersed foci that encircled the cell perimeter and became more abundant in the spermatocyte stages (Figure 1A,B). Fusomes are regions of actin-rich vesiculated cytoplasm that extend through intercellular bridges called ring canals, proposed to function in coordinating male germline cell division in early spermatogenesis (Hime et al., 1996; Wilson, 2005). Phalloidin-labeled fusomes were found to be abundant and highly branched in cysts containing spermatogonia, and less abundant and more globular-shaped in spermatocyte cysts; LGR1 immunoreactivity did not colocalize with fusomes in spermatogonia and spermatocyte stages (Figure 1A,B). In early spermatids, which are characterized with condensed, round-shaped nuclei, LGR1 immunoreactivity concentrates to single spots that are polarized to either end of the nuclei and, as nuclear elongation proceeds in late spermatids, receptor immunoreactivity is prominently detected as an elongated structure distinct from but remaining closely associated to the nuclei (Figure 1B,C). Upon completion of spermiogenesis, LGR1 immunoreactivity was not associated with spermatozoa, but rather detected in the cyst cytoplasm as punctate foci that are encapsulated within F-actin stained waste bags (Figure 1D) (Texada et al., 2008), which are organelles responsible for disposing of excess contents after sperm individualization (Metzendorf and Lind, 2010). LGR1 protein was found to stain basolateral surfaces of epithelia of the vas deferens and associated reproductive tissues in females (Figure 1E,F), but again, was not associated with spermatozoa in the vas deferens or the spermathecae of female mosquitoes (Figure 1E,F). Taken together, LGR1 immunolocalization (Figure 1A,F) along with the distribution of LGR1 transcript (Figure 1G,L), revealed expression associated with immature stages of sperm, but not in spermatozoa, suggesting that LGR1 may contribute toward coordinating the development of sperm.

**FIGURE 2:**
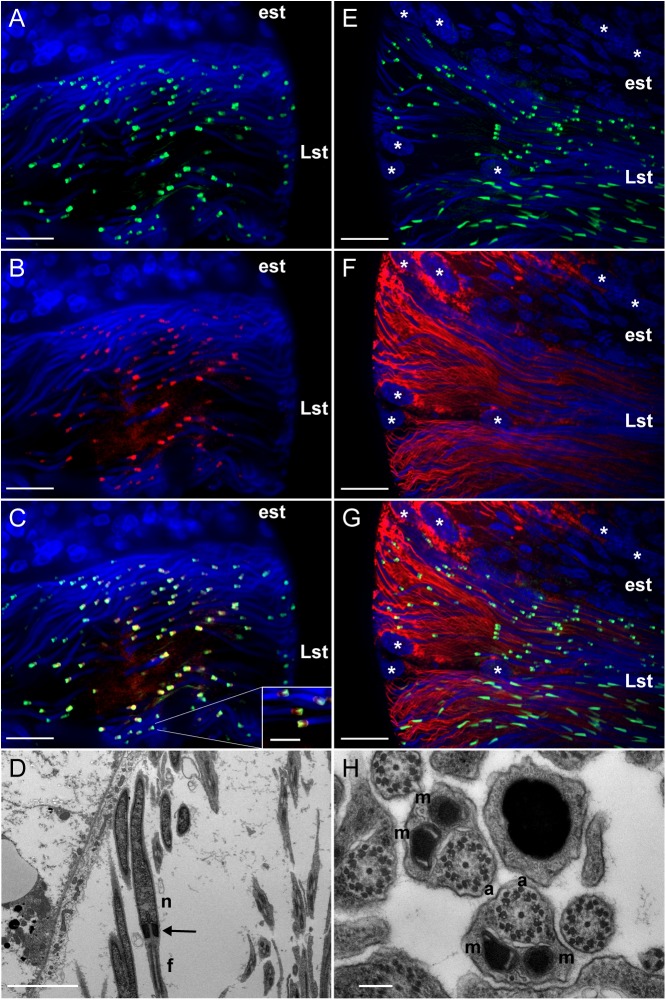
Distinct localization of LGR1 immunoreactivity in late-staged spermatids of adult *A. aegypti* testes. **(A,C,E,G)** Testes were stained with anti-LGR1 (green), **(B,C)** anti-gamma tubulin (red) as a marker for the centriole adjunct (Dallai et al., 2016), **(F,G)** MitoTracker (red) to detect mitochondrial derivatives in flagella, and nuclei (blue). **(C)** LGR1 immunoreactivity co-localizes with gamma-tubulin at the centriole adjunct. **(D)** Transmission electron microscopy (TEM) image showing the centriole adjunct (arrow) is positioned between the nucleus (n) and flagella (f) of spermatids. **(G)** LGR1 immunoreactivity is not found associated with the mitochondrial derivatives in the flagella of late-staged spermatids, comprised of **(H)** two mitochondrial derivatives (m) and an axoneme (a). Early staged spermatids (est), late-staged spermatids (Lst), cyst cell nuclei (^∗^). Scale bars: 10 μm in panels **A–C**, 5 μm in panel **D**, 20 μm in panels **E–G** and 200 nm in panel **H**.

In late spermatids, LGR1 immunoreactivity most prominently associated with an elongated region that attached to nuclei (Figure 1C). To structurally identify this elongated region, co-localization experiments were performed with markers that stain in the spermatid collar and tail regions. Results indicated LGR1 immunoreactivity was associated with the centriole adjunct in the collar region of developing spermatids, that was stained using an antibody targeting gamma tubulin (Dallai et al., 2016), rather than mitochondrial derivatives in flagella (Figure 2). The centriole adjunct is a region found between the nucleus and flagella of sperm (Figure 2D), and contains more than 100 proteins responsible for nucleating cytoplasmic microtubules, ultimately required for proper flagellar development during spermatid stages (Nigg and Raff, 2009; Dallai et al., 2016). Since LGR1 immunoreactivity was associated with this region in developing spermatids, we next sought to identify whether knockdown of LGR1 expression influenced (i) flagellar development, (ii) the quantity of spermatozoa and (iii) male fertility.

### Bacteria-Mediated LGR1 Knockdown

**FIGURE 3:**
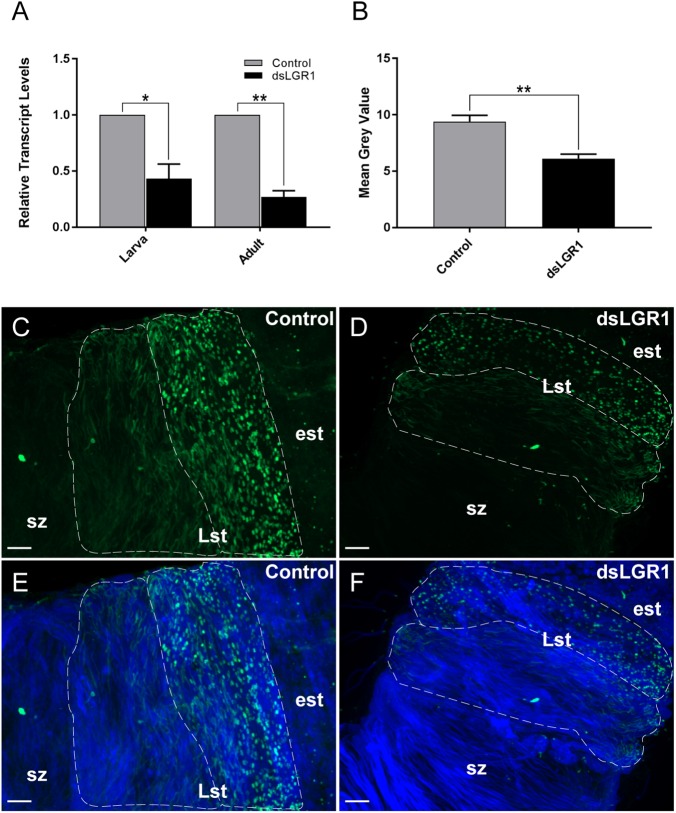
LGR1 knockdown efficiency in adult *A. aegypti*. Second instar larvae were fed with *Escherichia coli* expressing LGR1 dsRNA or control dsRNA (empty L4440 vector). After feeding, LGR1 transcript knockdown efficiency was determined in panel **(A)** whole 4th-instar larvae and one-day old adults by quantitative RT-PCR. LGR1 immunoreactivity was examined in control and LGR1 knockdown treatments within **(B–F)** the testes of virgin one-day adult males (LGR1, green; nuclei, blue). **(A)** LGR1 transcript levels of 4th instar larvae and newly emerged adults fed with LGR1 dsRNA were significantly reduced compared to control dsRNA-fed mosquitoes. **(B)** Quantification of LGR1 immunoreactivity from control and dsLGR1-treated larvae confirms downregulation. Representative confocal images demonstrate reduced LGR1 immunofluorescence in cysts (dotted outline) containing late-staged spermatids (Lst) when comparing **(C,E)** control and **(D,F)** dsLGR1-treated mosquitoes. All data are presented as mean ± SEM (*n* = 3 in panel **A**, *n* = 11–13 in panel **B**) with asterisks representing significant differences between control and dsLGR1-treated mosquitoes as determined using an unpaired *t*-test: ^∗^*P* < 0.05, ^∗∗^*P* < 0.01. Early staged spermatids (est), spermatozoa (sz). Scale bars: 20 μm.

RNA interference of the *A. aegypti* LGR1 gene was accomplished using a bacteria-mediated gene-silencing mechanism established previously (Whyard et al., 2015). Larvae were fed RNAse III-deficient HT115 *E. coli* (Timmons et al., 2001) producing LGR1-targeted or control (no insert) dsRNA. Relative to control mosquitoes fed with bacteria containing dsRNA derived from non-recombinant pL4440 plasmid, LGR1 transcript was significantly reduced by ∼57% in whole fourth-instar larvae and ∼73% in newly emerged (<1 day old) adults (Figure 3A). Moreover, LGR1 protein expression in the testes of newly emerged adults, assessed by comparing the intensity of LGR1 immunoreactivity in cysts containing spermatids, was significantly decreased in dsLGR1-fed mosquitoes compared to controls (Figure 3B–F).

### LGR1 Knockdown Influences Spermatozoa Count, Flagellar Morphology and Male Fertility

**FIGURE 4:**
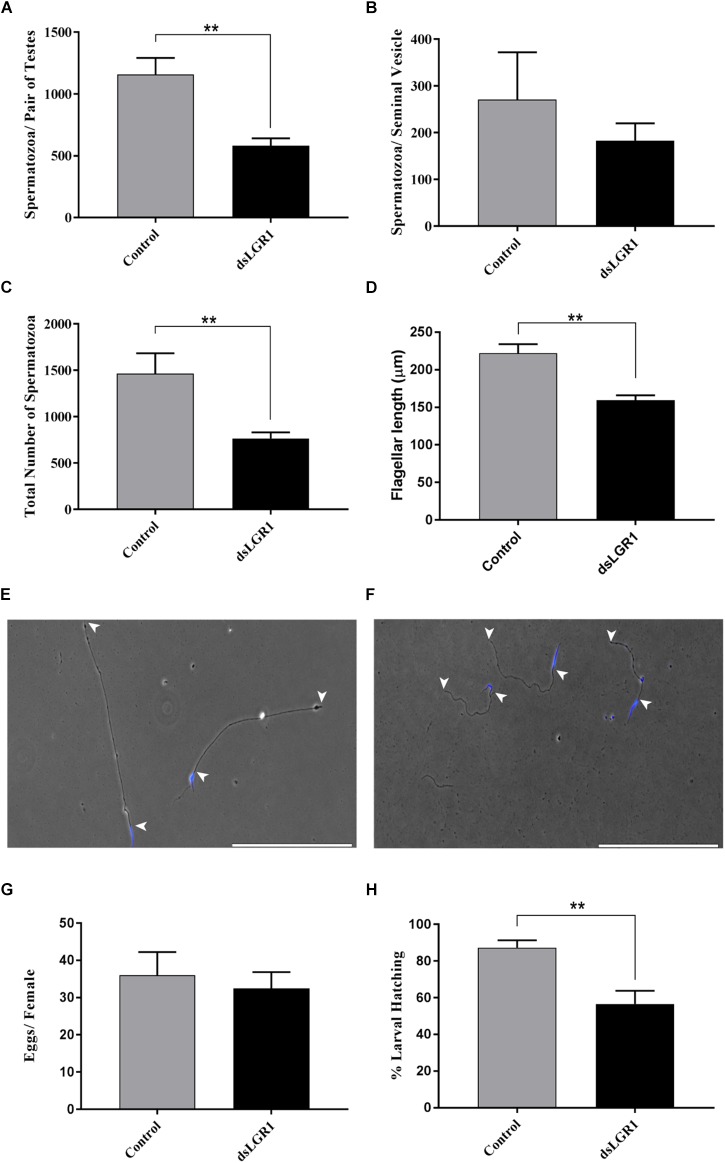
Effects of LGR1 knockdown on spermatozoa yield, flagellar length and reproductive success of adult male *A. aegypti*. Second instar larvae were fed with *E. coli* expressing LGR1 dsRNA or control dsRNA (empty L4440 vector) and the effects of knockdown on sperm yield, flagellar length and male fertility were analyzed in adult stages. Relative to controls, **(A–C)** dsLGR1-fed mosquitoes had significantly less spermatozoa in testes and in the overall reproductive tract (testes + seminal vesicle), while the number of spermatozoa in the seminal vesicle showed a similar trend but was not significant. **(D)** Flagellar lengths from spermatozoa of dsLGR1-treated mosquitoes were significantly shorter than flagella from control mosquitoes. Representative images showing flagellar lengths (arrowheads indicate beginning and end of flagellum) of spermatozoa collected from **(E)** control- and **(F)** dsLGR1-treated mosquitoes (nuclei, blue). **(G,H)** No differences in egg yield were observed between females mated with control or dsLGRl-treated males; however, the percentage of larvae hatched from eggs was significantly reduced in matings that involved male LGR1 knockdown treatments. All data are presented as mean ± SEM (*n* = 17 in panel **A–C**, *n* = 14–15 in panel **D**, *n* = 13 in panel **G,H**) with asterisks representing significant differences between control and dsLGR1-treated mosquitoes as determined using an unpaired *t*-test: ^∗∗^*P* < 0.01. Scale bars: 200 μm.

Sperm was collected separately from the testes and seminal vesicles of newly emerged adult virgin *A. aegypti* males and the quantity of mature spermatozoa was compared between mosquitoes fed with *E. coli*-expressing LGR1-targeted dsRNA or *E. coli* expressing dsRNA derived from a non-recombinant pL4440 plasmid. For spermatozoa collected from testes samples, LGR1 knockdown resulted in a significant reduction in the number of spermatozoa compared to controls (Figure 4A) and a similar trend was observed for spermatozoa isolated from seminal vesicles (Figure 4B), although this difference was not significant. Overall, when examining the total amount of mature sperm present in the male reproductive system, LGR1 dsRNA-treated mosquitoes possessed an average of 60% less mature spermatozoa than controls (Figure 4C). Testes-derived spermatozoa were then further analyzed for differences in flagellar morphology to identify if LGR1 knockdown had any other effect beyond the significant reductions in quantity of mature spermatozoa. The average length of flagella from control mosquitoes was ∼220 μm, whereas in LGR1 knockdown mosquitoes, the average flagellar length was reduced by ∼28% averaging ∼159 μm (Figure 4D–F).

To examine whether these effects on spermatogenesis led to any appreciable changes to male reproductive success, males fed on control or dsLGR1-producing bacteria were individually mated with untreated 6-day old virgin females. After the engorgement of a blood meal, egg batches from each female were counted, and the percentage of larvae that emerged was recorded. There was no significant difference in the number of eggs oviposited by females mated with either control or dsLGR1-treated males; notably, however, there was a significant reduction (∼30%) in the number of larvae that emerged from eggs laid by females mated with dsLGR1 males compared to control-treated males (Figure 4G,H).

## Discussion

Reproduction is tightly regulated by hormones in both vertebrate and invertebrate organisms. In female mosquitoes, oogenesis (the process of egg development) is coordinated by several well-characterized hormonal signaling pathways such as juvenile hormone, ecdysteroids, ovary ecdysteroidogenic hormone, and insulin-like peptides (Raikhel and Lea, 1990; Brown and Cao, 2001; Raikhel et al., 2005; Gulia-Nuss et al., 2012; Dhara et al., 2013; Hansen et al., 2014; Gulia-Nuss et al., 2015). In males, some evidence for endocrine regulators of spermatogenesis is present in the fruit fly *Drosophila melanogaster* (Kiger et al., 2000; Ueishi et al., 2009; Schwedes et al., 2011). However, research directed toward understanding how spermatogenesis is hormonally regulated in mosquitoes remains unexplored. A previous study examining the expression profile of the glycoprotein hormone receptor LGR1 in *A. aegypti* showed transcript enrichment and LGR1 immunoreactivity in male and female reproductive organs, indicating that its ligand GPA2/GPB5 may encompass a reproductive function (Rocco et al., 2017). In the female ovaries, LGR1 was found expressed in follicular epithelia that house oocytes and nurse cells that aid in egg development (Rocco et al., 2017), whereas in the testes of male mosquitoes, LGR1 immunoreactivity localized to distinct areas of developing sperm (Rocco et al., 2017). Given its confirmed expression at the transcript level and regionalized immunoreactivity in the testes, the current study aimed to elucidate the physiological role of LGR1 in *A. aegypti* spermatogenesis.

### LGR1 as a Regulator of Microtubule Nucleation in the Spermatid Centriole Adjunct

LGR1 immunoreactivity was most apparently associated with an elongated structure positioned at the base of spermatid nuclei, which co-localization experiments identified as the centriole adjunct (Figure 1, 2). In developing spermatids, the centriole adjunct is a cylindrical sheath that forms around the centriole, that functions to anchor the flagellar axoneme (Dallai et al., 2016). Associated with the centriole adjunct is electron-dense pericentriolar material which consists of cytoplasmic proteins, such as gamma-tubulin and CP190, that are responsible for nucleating microtubules to form the flagellar axoneme (Riparbelli et al., 1997; Dallai et al., 2016). Experiments that aimed to elucidate the subcellular distribution of LGR1 immunoreactivity during spermatogenesis revealed LGR1 immunoreactivity associates with the cell perimeter (i.e., plasma membrane) of spermatogonia and spermatocytes (Figure 1A,B), which parallels previous findings that *A. aegypti* LGR1 is a membrane-associated receptor (Rocco et al., 2017). In late-staged spermatids, LGR1 staining is associated with the plasma membrane and regionalized to the centriole adjunct given that receptor staining encapsulates gamma-tubulin staining, which is a cytoplasmic protein. Taken together, it is hypothesized that LGR1 is localized to the membrane of the centriole adjunct where it is exposed and can be activated by its known ligand GPA2/GPB5 to coordinate microtubule nucleating activity in this region in spermatids. Interestingly, strong GPB5 immunoreactivity is associated with rat oocytes (Sun et al., 2010) and GPB5 transcript in previtellogenic-staged oocytes in *Amphioxus* (Tando and Kubokawa, 2009), which supports the hypothesis that GPA2/GPB5-LGR1 signaling may be involved in gamete development in mosquitoes.

Microtubule nucleating activity in the centriole adjunct is transient, given that staining for gamma tubulin and other pericentriolar proteins disappear when mature spermatozoa are formed (Riparbelli et al., 1997; Dallai et al., 2016). LGR1 transcript and immunoreactivity was detected throughout early stages of spermatogenesis and late stages of spermiogenesis, but not in mature spermatozoa. The receptor immunoreactivity was localized to waste bags, which are involved in removing excess cellular material following spermiogenesis (Arama et al., 2003; Fabian and Brill, 2012; Ben-David et al., 2015) (Figure 1). Given its close localization with gamma-tubulin in spermatids and transient expression pattern during spermatogenesis, we tested whether LGR1 is involved in microtubule nucleation and, consequently, flagellar development.

### LGR1 as a Regulator of Spermatogenesis

Depending on the mosquito species, spermatogenesis predominantly occurs in late larval to pupal developmental life stages (Lum, 1961; Barr, 1974). As a result, to assess the influence of LGR1 knockdown on spermatogenesis in *A. aegypti*, *E. coli* bacteria expressing LGR1-targeted dsRNA were fed to 2nd instar larvae. The effectiveness of LGR1 knockdown was then measured throughout mosquito developmental stages, as well as in the testes of adult males, and measured relative to control mosquitoes fed with *E. coli* expressing dsRNA from a non-recombinant pL4440 plasmid (Figure 3). Results showed that LGR1 gene knockdown was maintained to adulthood, where LGR1 transcript was significantly downregulated by ∼73%. Moreover, in the testes of adults, LGR1 immunoreactive staining intensity in spermatid-containing cysts was decreased by ∼35% relative to the cysts of control testes (Figure 3).

LGR1 knockdown mosquitoes were examined for abnormal phenotypes associated with spermatogenesis including morphological defects as well as differences in the quantity of spermatozoa from reproductive organs since defective sperm likely undergo apoptosis and/or may be targeted for degradation (Sutovsky et al., 2001; Baum et al., 2005). Relative to control mosquitoes, LGR1 knockdown led to a significant reduction in the quantity of mature spermatozoa, and the remaining spermatozoa were characterized with significantly shorter flagellar lengths (Figure 4). Given the impact of LGR1 knockdown on *A. aegypti* spermatogenesis, we next sought to determine whether this had any consequences on the reproductive success of male mosquitoes. Interestingly, a significantly reduced proportion of eggs hatched when females were mated with LGR1 knockdown males compared to control males (Figure 4). This could be interpreted as a result of abnormalities in sperm quality and reduced quantity from LGR1 knockdown males, which displayed a lower number of mature spermatozoa, and of the remaining spermatozoa, flagellar morphological defects were evident.

## Concluding Remarks

In summary, our data provide evidence for the involvement of LGR1 in mosquito spermatogenesis, given that knockdown of this receptor decreases sperm yield, impairs flagellar morphology and renders males less fertile. Specifically, our data indicates that LGR1 functions to regulate flagellar development during spermatogenesis since LGR1 immunoreactivity was regionalized to the centriole adjunct of spermatids, a region that houses proteins important for producing the axoneme. Given that LGR1 is the receptor target of the glycoprotein hormone GPA2/GPB5 (Sudo et al., 2005), our results support a reproductive function for GPA2/GPB5 in the mosquito. To our knowledge, this is the first study that examines endocrine signaling in the regulation of reproductive processes in male *A. aegypti*. In past studies, expression profiling of the allatotropin and allatostatin neuropeptide receptors identified transcript enrichment in the testis (Mayoral et al., 2010; Nouzova et al., 2012), suggesting that alternate endocrine signaling pathways exist and may also control sperm development in mosquitoes. Though most research allocated to understanding mosquito biology has been directed toward female reproduction, male reproductive biology is understudied and should be considered in future research. Research focused on better understanding the reproductive biology and its endocrine of male mosquitoes will be vital for the development of novel approaches and the improvement of existing pest control strategies aimed at lessening the burden of these medically important vectors of disease.

## Author Contributions

DR and J-PP contributed to the experimental design with feedback from RN and wrote the paper with revisions made by RN, AG, ES, and DdS. DR, AG, ES, and DdS performed the experiments. DR, AG, ES, DdS, RN, and J-PP aided with data analysis.

## Conflict of Interest Statement

The authors declare that the research was conducted in the absence of any commercial or financial relationships that could be construed as a potential conflict of interest.
