# Supplementary material for: Glycoprotein Hormone Receptor Knockdown Leads to Reduced Reproductive Success in Male Aedes aegypti
Source: Front Physiol. 2019 Mar 19;10:266. doi: 10.3389/fphys.2019.00266 (PMC6433794; doi:10.3389/fphys.2019.00266)
Supplement: Supplementary file 1 [file Table_1.DOCX]

Supplementary Material

Table S1. Primers utilized for RNA probe template generation, bacteria-mediated RNA interference dsRNA target design, and quantitative PCR analysis of LGR1 in *Aedes aegypti* (Genbank accession number: KF711859). Positions of oligonucleotides refer to the LGR1 full open reading frame as reported previously (1).

| Oligonucleotide Name: | Oligonucleotide Sequence: | Product size (bp) | Nucleotide position (exons, base number): | Application: |
| --- | --- | --- | --- | --- |
| LGR1-F | TGTTAAGTGCTACCCGATGC | 610 | 11-13, 1608-2218 | RNA probe generation for fluorescence *in situ* hybridisation |
| LGR1-R | TGACGATGATGATGAAGGCC |  |  |  |
| T7 Promoter | TAATACGACTCACTATAG |  |  |  |
| LGR1-F | ATGCGAGGACGTAATGGGAT | 735 | 11-13, 1644-2378 | dsRNA Target Sequence for bacteria-mediated RNA interference |
| LGR1-R | ATGGCTGTCAGTCCGAAGAA |  |  |  |
| LGR1-F | GCCGGTTGCGTATCTTTTC | 290 | 9-11, 1319-1608 | Quantitative PCR |
| LGR1-R | ATCAAATGTTGTGGGCGTAAG |  |  |  |
| rp49-F | ACAAGCTTGCCCCCAAC | 214 |  |  |
| rp49-R | GCGATTTCGGCACAGTAGA |  |  |  |
|  |  |  |  |  |

**References**

Paluzzi JP, Vanderveken M, O’Donnell MJ (2014) The heterodimeric glycoprotein hormone, GPA2/GPB5, regulates ion transport across the hindgut of the adult mosquito, *Aedes aegypti*. *PLoS One* 9(1):1–14. https://dx.doi.org/10.1371%2Fjournal.pone.0086386
